# Immersive participatory design of assistive robots to support older adults

**DOI:** 10.1080/00140139.2024.2312529

**Published:** 2024-02-14

**Authors:** Samuel A. Olatunji, Vy Nguyen, Maya Cakmak, Aaron Edsinger, Charles C. Kemp, Wendy A. Rogers, Harshal P. Mahajan

**Affiliations:** a College of Applied Health Sciences, University of Illinois Urbana-Champaign, Urbana, IL, USA; b Research and Development, Hello Robot Inc., Martinez, CA, USA; c School of Computer Science and Engineering, University of Washington, Seattle, WA, USA

**Keywords:** Human robot interaction, ethnographic research, ageing in place, user centred design, assistive technologies

## Abstract

Assistive robots have the potential to support independence, enhance safety, and lower healthcare costs for older adults, as well as alleviate the demands of their care partners. However, ensuring that these robots will effectively and reliably address end-user needs in the long term requires user-specific design factors to be considered during the robot development process. To identify these design factors, we embedded Stretch, a mobile manipulator created by Hello Robot Inc., in the home of an older adult with motor impairments and his care partner for four weeks to support them with everyday activities. An occupational therapist and a robotics engineer lived with them during this period, employing an immersive participatory design approach to co-design and customise the robot with them. We highlight the benefits of this immersive participatory design experience and provide insights into robot design that can be applied broadly to other assistive technologies.

## Introduction

### Meeting care-related needs of an ageing population

Many countries are facing a significant care gap where the number of older adults (aged 60 years and older) is rapidly growing without an equivalent growth in potential support ratio (Miyamoto, and Yoshino, 2022). For instance, in 2020, the older adult population out-numbered children younger than five years (World Health Organization 2022). At this growth rate, there may likely be older adults by 2050 who require the support of a younger population who are insufficient in physical number and resource capacity to manage the prevailing care demands (World Health Organization 2022). However, the older adult population is not monolithic. Some older adults within the most senior group of older adults (85 years or over) have intact physical, cognitive, and perceptual abilities and can live independently. Conversely, there are older adults within the youngest-old group (65 years to 74 years) who have experienced significant declines in capacities and are dependent on support to carry out activities of daily living (Czaja et al. 2019). Understanding this variability in the demographic spectrum is critical for developing a comprehensive public health response and support strategies including the use of technology. This is to address the wide range of older people’s needs to ensure they can enjoy health and age successfully.

Ageing in place can be defined as ‘One’s journey to maintain independence in one’s place of residence as well as to participate in one’s community’ (p. 1, Rogers, Ramadhani, and Harris 2020). Achieving this goal will depend on providing individuals with physical, psychological, and psychosocial support to maintain their independence and autonomy. Some older adults encounter challenges and/or difficulties that impede the ease of living independently, such as falls or physical impairments, which lead to more care-related health and safety concerns (Beer et al., 2017). This often results in the need for many older adults, particularly those with no family care partners, to be moved into assisted care and residential facilities that are rapidly running into caregiving staff shortages (Chu, Yee, and Stamatopoulos 2022). Keeping older adults safe and functional at home can save long-term healthcare costs and help maintain a good quality of life. Data suggests that an estimated $1.1 billion could be saved annually for every additional month that individuals can remain independent (Davey et al. 2004; Johnson, Davis, and Bosanquet 2000; Van Leeuwen et al. 2019).

This foregrounds the role that assistive robotic technologies could play in supporting older adults ageing in place. There are a variety of robots already commercialised or in the development phase, designed to help with or perform home tasks (Ostrowski et al., 2019). Such robots are designed to provide physical support (e.g. robots for house chores, Beer et al. 2012), cognitive stimulation (e.g. robots supporting MCI patients, Law et al. 2019), social communication (e.g. social companion robots, Wada et al. 2004), and safety monitoring (e.g. Robots supporting wellness check robots, Smarr, Fausset, and Rogers 2011). Older adults are open to using these robots for various applications at home (Ezer et al., 2009). However, despite the positive attitude towards using these robots, there remains a significant gap between the potential of these robots and the rate of acceptance by older adults for long-term use. One reason could be a lack of adequate consideration for the overall goals, challenges, abilities, tendencies, concerns, and preferences of older adults during the design process.

Our review of the development of assistive robots revealed few studies that collectively examined these design factors. A feasible strategy to identify these fac tors is to include the potential end-users (i.e. older adults) in the design team to better represent their preferences in the design. In this paper, we propose a method of achieving this called *immersive participatory design*. We provide an exemplary case study of the method and discuss the inherent benefits of advancing the design of user-centred assistive robots.

### Designing assistive robots to support older adults

When designing technology to support independence and ageing in place, stakeholders from multiple fields should consider the interaction of person, space, and time aspects of ageing in place (Rogers, Ramadhani, and Harris 2020). This involves a vivid awareness of the person’s needs, abilities, capacity, and experiences and how these change over time. These are precursors that offer tacit knowledge needed for the design of technology that fits the lifestyle of the potential user. These also provide an allowance for adaptations that may be required to meet the changing demands of everyday needs. This is a major value derived from participatory design, a process that involves end-users in the design process. Co-design is an integral component of participatory design, and it can be described as the active collaboration between the stakeholders in designing solutions to a prespecified problem (Vargas et al. 2022). In this context, the process provides the opportunities to harness the experiences, capabilities, limitations, and preferences of older adults for the design of robots that will support successful ageing (Rogers, Kadylak, and Bayles 2022).

The immersive experience involves conducting research in the users’ environment, where the stakeholders and researchers are deeply engaged in the design process. This inspires a more comprehensive understanding of the peculiarities, complexities, and constraints in the user’s environment. Combining the immersive experience with participatory design yields an design method where the outcome effectively meets the users’ needs (Table 1). This approach is the core value presented in this research – a value that we envision has the potential to make the goal of successful ageing feasible with assistive robots. Our aim is to ensure that older adults can use these robots in the way they want, when they want, with whom they want, where they want, and how they want (Lee and Riek 2018; Rogers, Ramadhani, and Harris 2020).

The focus of this paper is threefold: First, to introduce the immersive participatory design process as a user-centred approach to iterative development and evaluation. Second, to provide a case study to illustrate its utility for a use case with two key stakeholders represented – a care recipient and a care partner. We use an extreme case of an older individual with a high degree of motor impairments who is also an expert in human-robot factors. And third, to provide insights into the robot design process that can be applied broadly beyond this specific use case.

## Materials and methods

### Immersive participatory design process

The concept of immersing in an environment aligns with the goal of immersive learning (Strangman and Hall 2003), where visualisations of an environment experienced by a user absorbed into such an environment inspire a deeper understanding of the environment – its context, purpose, properties, and constraints (Bhattacharjee et al. 2017). Participatory design involves understanding the activities of individuals from their perspective to elicit underlying knowledge users have about these activities, why they do them, how they want to do them, and other elements of knowledge that will inform design (Spinuzzi, 2005). Immersing researchers and technology designers in the user’s environment can serve as a keystone to better harness the value that participatory design provides.

Bringing the immersive experience into participatory design leads to an in-depth active engagement of potential users in the design process along with the developers and researchers from the initial conception and formative evaluation of the product to the summative evaluation. As an example, potential users can actively participate as co-designers throughout the entire design process, ensuring that the product’s design not only meets their needs but also enhances their abilities and addresses their concerns. This intersection between immersion and participatory design principles would involve a mix of quantitative, qualitative, and ethnographic approaches. The purpose is to evoke knowledge, reasons, purpose, and experience from all individuals involved in the design process, resulting in a holistic design that meets real-world needs. We refer to this process and technique as immersive participatory design.

In the design of assistive robots, immersive participatory design plays a significant role in ensuring the task needs and preferences of the potential users are adequately and promptly addressed during the robot design process. For example, the designers and researchers can be actively involved with the older adults in the design process through iterative co-active design sessions leading to user-centred designs that fit the mental model of the user regarding the use, role, and functionalities of the resultant robot design. This study exploits the value derived from this approach, leading to an assistive robot design that has the potential to support older adults in different activities in home environments.

### Use case: Care recipient (Henry Evans) and care partner (Jane Evans)

We present an immersive participatory design journey we undertook in the development of an assistive robot design that has the potential to provide support in various aspects of everyday tasks relevant to older adults. We begin the narration of this journey by introducing you to our case study dyad, Henry and Jane Evans, who stand out distinctly beyond the average potential user as they were actively involved in the assistive robot’s iterative design and formative evaluation.

In 2002, at the age of 40, Henry Evans experienced a brain-stem stroke secondary to a basilar artery dissection, causing him to be non-speaking with quadriplegia and requiring complete assistance from Jane to do the majority of his everyday activities. Since then, Henry and his wife, Jane Evans, have had extensive experience with emerging technologies, such as robotics, to promote greater independence for Henry (note that Henry & Jane permitted us to use their names in this paper). In addition to enabling Henry’s independence in his activities, we wanted to determine ways assistive robots can alleviate Jane’s care demands as a care partner to promote her participation in everyday activities. However, to ensure that users can successfully rely on such robots, appropriate design factors must be considered in their development. To identify these design factors, we embedded Stretch, a mobile manipulator created by Hello Robot, in Henry and Jane’s home for two cycles of two weeks to support them with their daily activities. This immersive participatory design session promoted the dyad to engage with Stretch while being supported by the research team members.

### The robot

**Figure 1.**
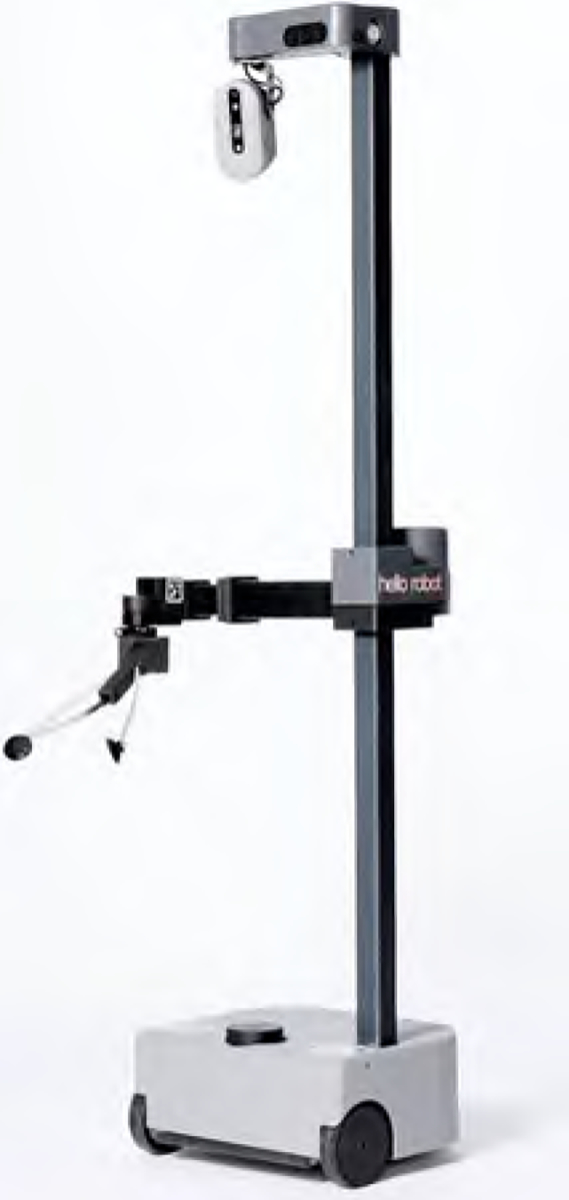
Stretch Research Edition robot (RE1) by Hello Robot (https://hello-robot.com/product).

The Stretch robot is a mobile robot manipulator designed to support everyday activities using a lightweight telescoping arm mounted on a mobile base (see Figure 1). The care recipient controlled Stretch via a web-based user interface on his computer using a head tracking device to move the mouse cursor and a mouse to click the interface buttons. The robot worked at two levels of autonomy: teleoperation level and semi-autonomous level. At the teleoperation level, the arm joints and mobile base of the robot are directly controlled through the web interface. At the semi-autonomous level, the action sequences of the robot arm and base could be captured as a user-defined function through the web interface. These actions can then be executed by the robot when needed, in the same sequence that the user captured the motions. This provides a form of user-centred automation mode through which the user can customise the motions of the robot to fit specific tasks as needed. The care partner interacted with Stretch using voice commands and an exercise program interface. There were design cycles where user testing was conducted, and feedback received from the participants was used to improve the developments. The immersive participatory design approach and cycles gave our interdisciplinary team the opportunity to review the findings and improve Stretch’s design, including the control components of the web interface.

Additional technical specifications of the robot are provided as follows:

- *Weight* – The overall mass of Stretch is 23 kg (50.5lb), with the majority of the mass in its base. The carbon fibre arm and aluminium mast create a remarkably lightweight upper body. This greatly reduces the level of imparted force the robot can generate during unexpected contact.
- *Contact Sensitivity* – The four primary joints of Stretch (base, lift, and arm) have contact sensitivity. We measure motor currents to estimate contact forces. Because Stretch is a low gear-ratio robot, current sensing provides a fairly sensitive measure of contact forces.
- *Firmware limits* – Motor torques were limited to the lowest level (0.1 Newton-Metres), which is a minimal rate of change of angular momentum that can be imparted with respect to the configured firmware bounds.
- *Velocity limits* – Fast motions of the base are restricted when the arm is up high and the tool is outside the base footprint. The robot’s maximum velocity was fixed at 0.2 metres per second for the base and 0.1 metres per second for the arm. This slow speed gives sufficient time for the researchers to intervene and stop the robot if necessary.
- *Tilt detection* – The robot can detect when its body is tilted beyond a safe threshold. The robot can be configured to trigger a run-stop event during an over-tilt event.

### Procedure

**Figure 2.**
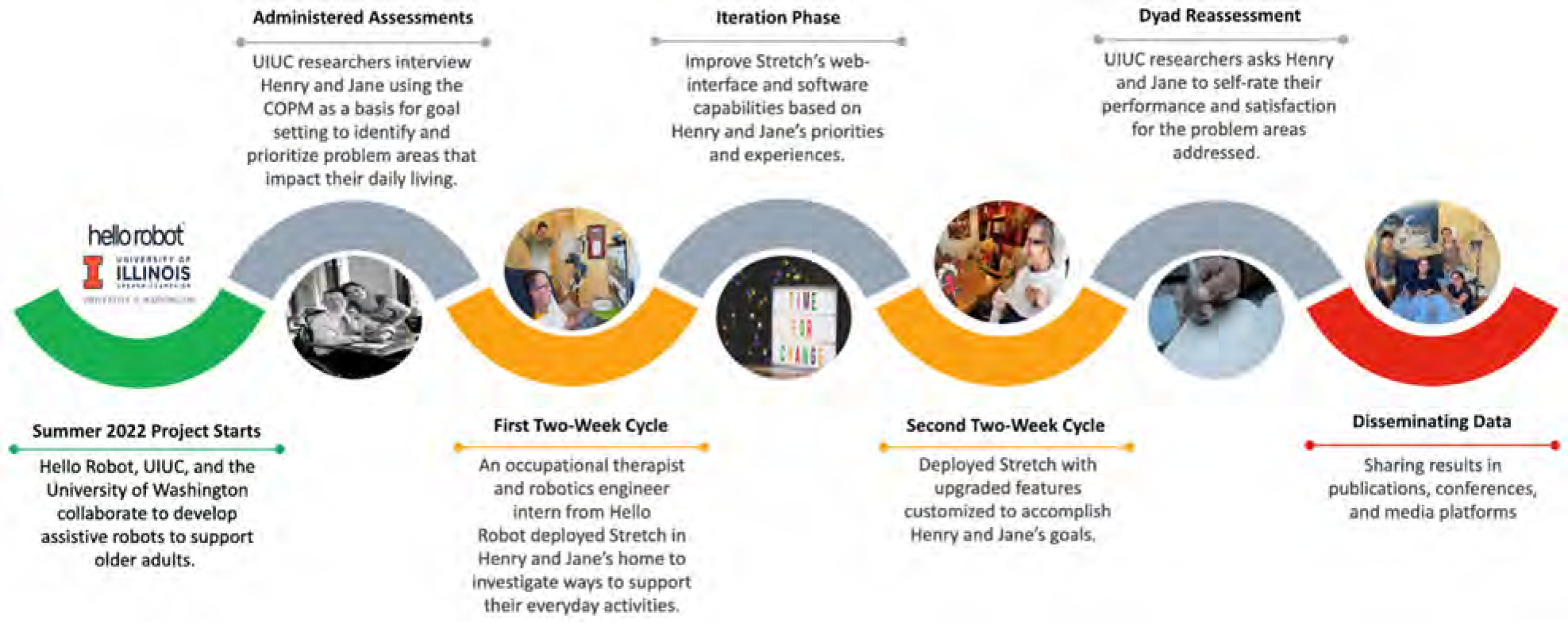
Flow of the study – the cycles and improvement between the cycles.

We embedded the robot, along with two of the Hello Robot staff (an occupational therapist (OT) and a robotics engineer), into the couple’s home to support their daily activities. Between each cycle of participatory design and evaluation, our interdisciplinary team, with expertise in robotics, occupational therapy, and human factors, reviewed the findings and improved Stretch’s design based on the feedback from the dyad. The study flow (provided in Figure 2) describes the activities carried out within the two two-week cycles. This study was approved by the Institutional Review Board of the University of Illinois Urbana-Champaign (protocol number – 23146).

**Figure 3.**
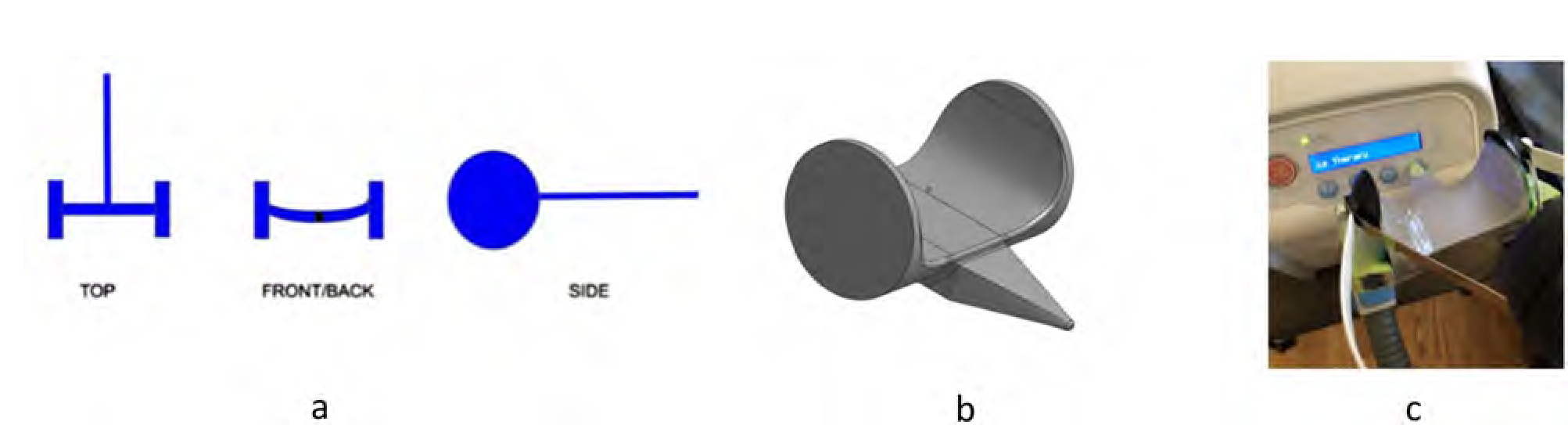
Henry’s design of the tool to operate his percussion vest machine to loosen mucus from his airway walls and improve airway clearance. (a) Henry’s drawing of the button pusher. (b) Computer-aided design of button pusher. (c) Henry uses Stretch’s gripper to grasp the 3D-printed button pusher and to start the button turn on the percussion vest machine.

**Figure 4.**
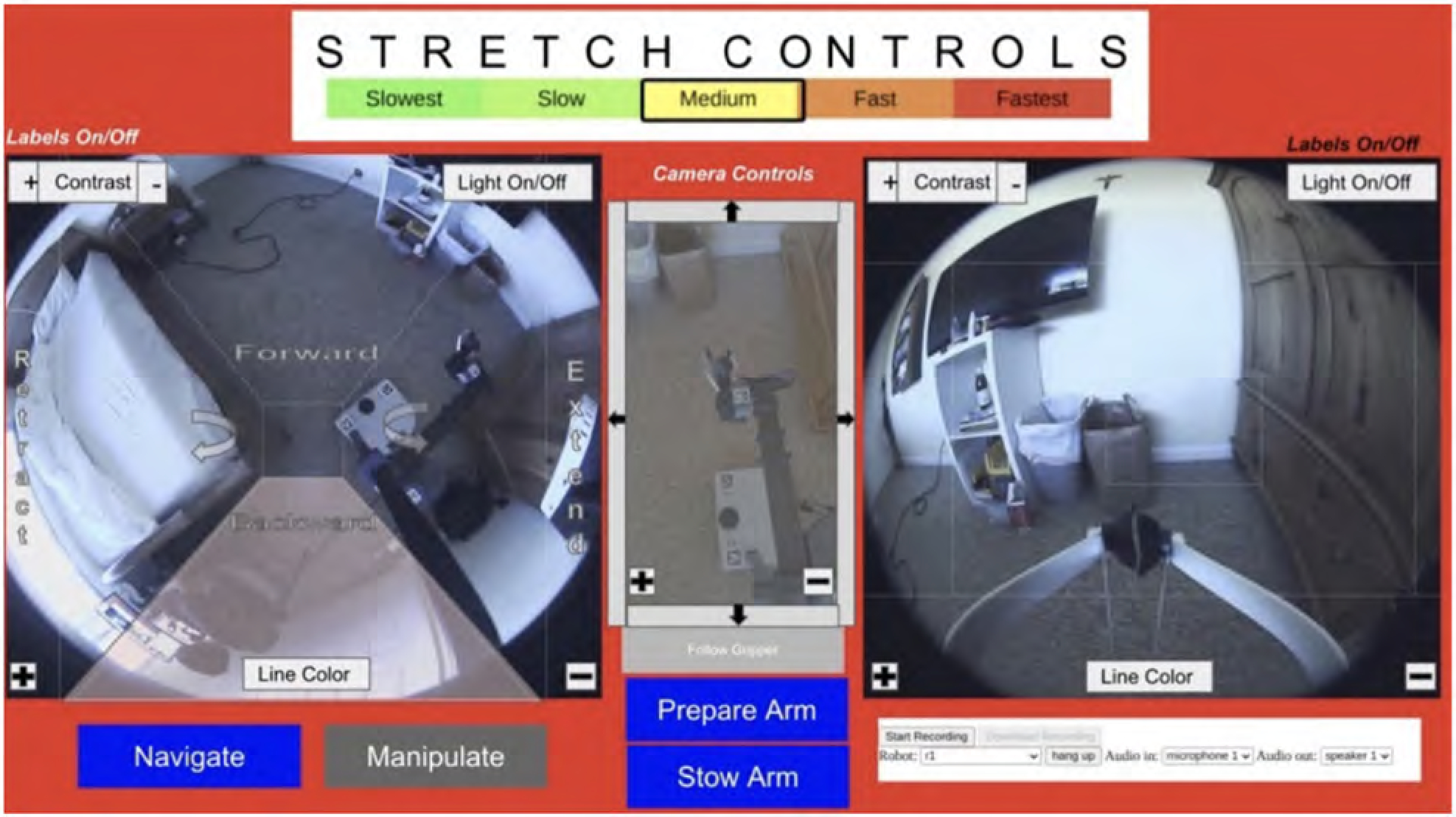
Henry’s web interface suggestions – with camera views to see different aspects of the robot’s task space.

Each cycle of participatory design and evaluation produced findings, feedback, and comments that needed to be addressed, leading to an improved version of Stretch’s design. Some of the design suggestions and drafts came from the care recipient who made sketches of end effector tools (example Figure 3) and web interface suggestions (example Figure 4), including ideas on shared autonomy for controlling the robot.

After the initial interviews were conducted to identify specific tasks that they would want Stretch to do in supporting them with these activities, both the care recipient and care partner used Stretch to perform the prioritised tasks they mentioned in the interviews. The immersive participatory design process allowed the Hello Robot team to identify tools that could be useful as attachments to Stretch in the home environment, which would make it more effective and efficient. They developed functional prototypes of these necessary tools for Stretch during their stay in the home and the intervals between cycles. The opportunity to stay within the home, observing the environmental variables, task constraints, and overall flow of activities within the day aided the planning and conceptualising of useful tools for the robot. This led to refinements and the creation of features that better supported the ease of use and reliability of the participants’ interactions with Stretch. The perceptions of the care partner and care partner were then evaluated through interviews, questionnaires, and on-site observation to obtain more feedback for the iterative process of development.

### The tasks

We used the Canadian Occupational Performance Measure (COPM, Law et al. 1990) to conduct a semi-structured interview that facilitated an open conversation with the care recipient and care partner to identify, prioritise, and evaluate issues in the important activities they encounter in their lives (Law et al. 2015). The COPM is administered as a questionnaire to identify priority tasks for the participants in the areas of self-care, productivity, and leisure. It assesses the level of current performance and satisfaction of the participants before and after the intervention. The COPM is based on the Canadian Model of Occupational Performance and Engagement (CMOP-E), an occupational therapy model in which a person-centred approach and enablement of everyday activities are core elements (Law et al. 2015). It encompasses the interaction between a person (with performance capabilities and spirituality), the occupations the person in the environment performs in the three components of self-care, productivity, and leisure, and the interaction with the environment (social, cultural, physical, institutional environment of the person) (Law et al. 2015). With the COPM, users can identify their important daily activities, facilitating a user-centric approach, including partnership and collaboration between the researcher and person (Wressle et al. 2002; Hansen et al. 2018; Enemark Larsen, Rasmussen, and Christensen 2018). In occupational therapy, addressing the client’s specific concerns and priorities enhances the effectiveness of interventions and contributes to their overall satisfaction with the therapeutic process.

**Figure 5.**
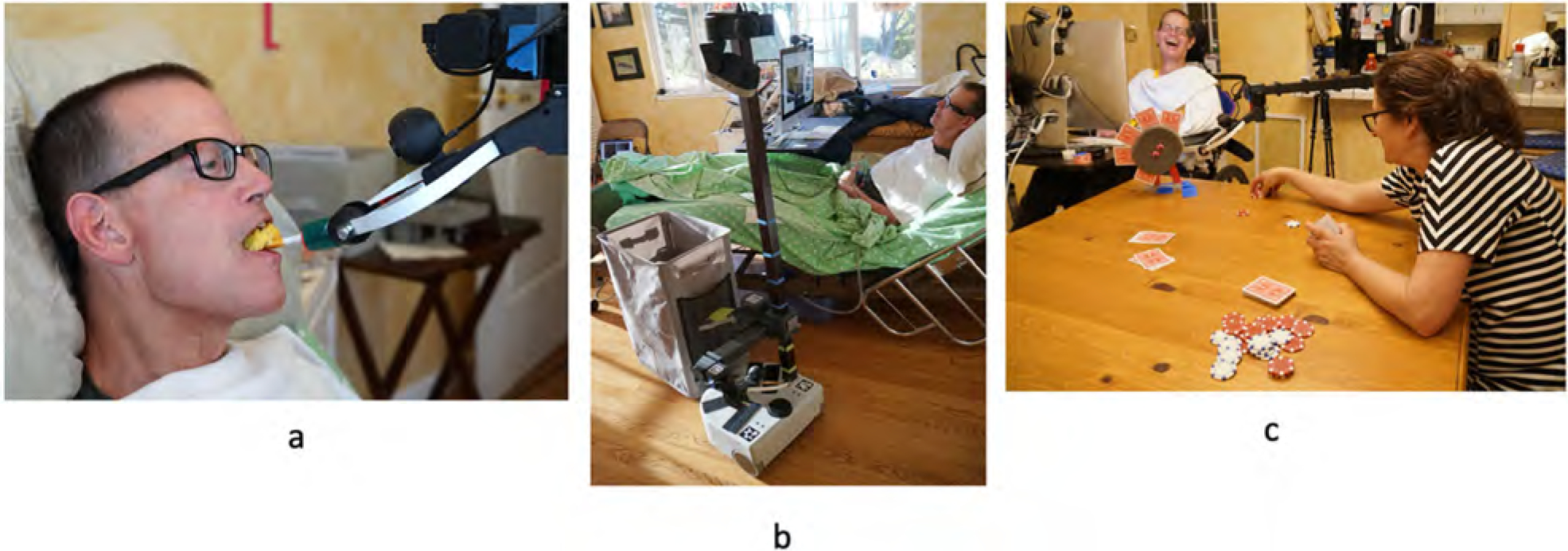
Examples of robot task support. (a) Henry self-feeds soft, pureed foods using a built-up silicone spoon. (b) Henry uses a magnetic towing mechanism on Stretch to tow a rolling laundry basket near the laundry room. (c) Henry uses a customised card holder to play poker with Jane.

Using the COPM process as a basis for goal setting, we narrowed down the problem areas in the aspects of self-care, productivity, and leisure to the five most important main tasks for the care recipient and four tasks for the care partner. We identified tasks based on the degree of importance that the care recipient and care partner associated with those tasks. The prioritised tasks for the care recipient were self-feeding, maintaining comfort, flipping a light switch, meal preparation and clean-up, and social participation through a group game. The prioritised activities for the care partner were physical exercise, meal preparation, household cleaning, meal preparation, assistance with laundry, and social participation with the care recipient (Highlights of these tasks are presented in Figure 5). The feasibility of using the Stretch robot to accomplish the tasks was then considered before finalising the selected tasks.

Henry performed most of his activities with setup assistance from the OT while providing feedback to ensure the Stretch and the tools were placed in his preferred optimal position. Henry participated in card games, such as poker, with Jane and their friends, which enhanced his social-emotional well-being and his self-efficacy of being on his own team. Henry’s increased independence with doing his daily activities enabled his care partner to participate in her leisurely activities, such as hiking and exercising, which were often unobtainable due to her care demands. The care recipient also played an active role in assisting his care partner with household tasks, such as towing a laundry basket and meal preparation, delivery, and clean-up, which restored his role of helping his wife with these shared responsibilities as he previously did prior to having a stroke.

## Results

As an interview-based measure and self-evaluation of performance and satisfaction with performance, the COPM offers high validity and reliability for stroke patients (Cup et al., 2003). It also defines the care partner’s perception of their own performance in their daily activities. As depicted in the Appendix, Henry and Jane shared their qualitative responses on how well Stretch supported them in accomplishing each of their prioritised tasks (marked as a COPM goal). The two cycles of exploration allowed Henry and Jane, as end-users, to play an essential role in contributing to the improvements of Stretch that best meet their needs and increased their success in accomplishing the activities that are most meaningful to them.

### Performance and satisfaction after intervention

**Figure 6.**
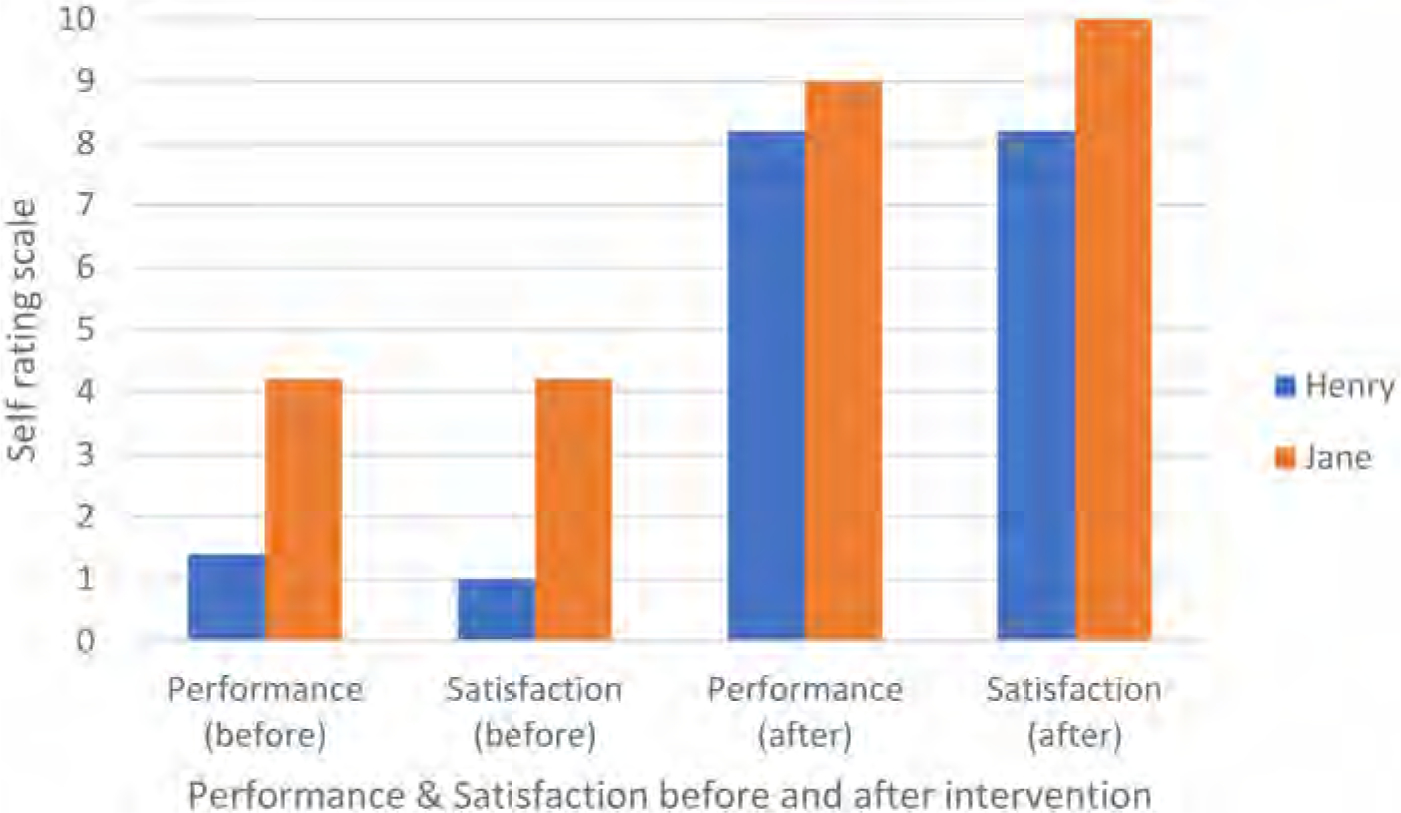
Average COPM Performance and satisfaction scores of participants before and after intervention with Stretch (Performance was rated as 1 = not able to do it all to 10 = able to do it extremely well. Satisfaction was rated as 1 = not satisfied at all to 10 = extremely satisfied).

A rating of performance (how the participants would rate the way they did the activity) and satisfaction (how satisfied the participants were with the way they did the activity) was conducted before and after the intervention with Stretch (see Figure 6). These scores were calculated as averages of ratings provided by Henry for the five tasks he carried out with Stretch and averages of ratings provided by Jane for the four tasks she carried out with Stretch. Henry’s performance improved by 68%, and his satisfaction increased by 72%. Jane’s performance also increased by 48%, and her satisfaction improved by 58%.

### Person factors and robot usability

**Figure 7.**
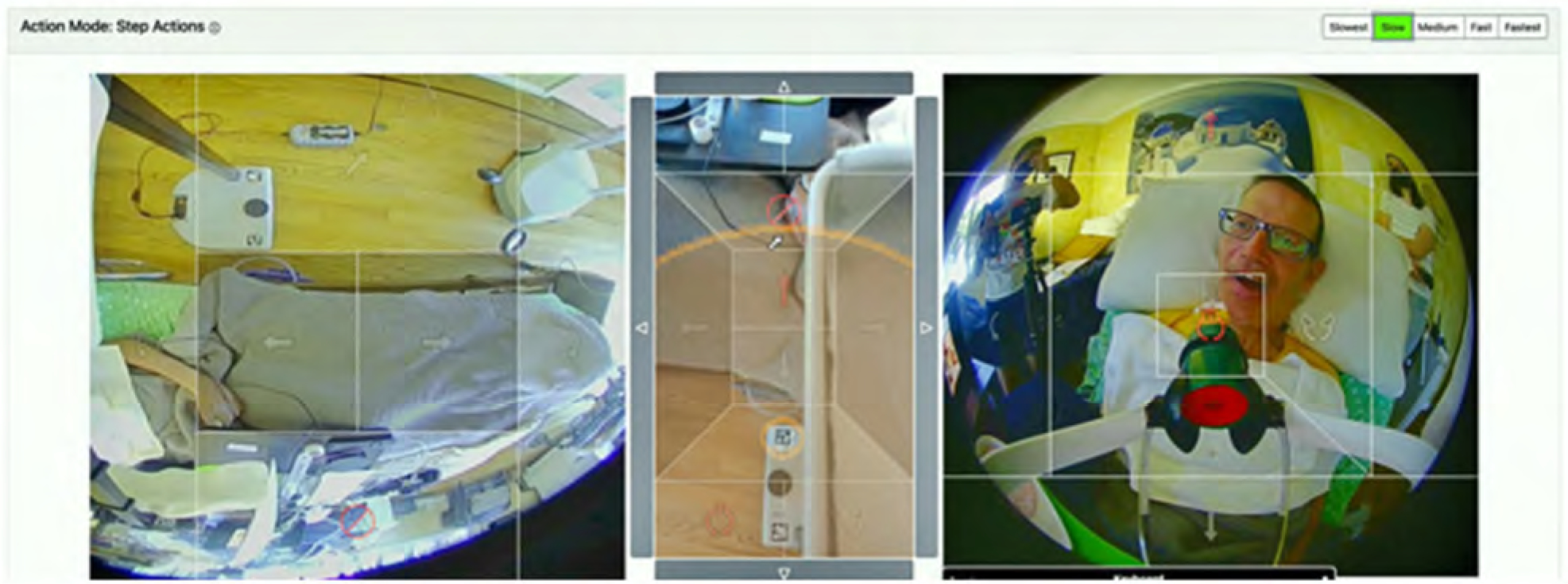
Image of Henry self-feeding using the initial web interface to operate Stretch.

Henry used a web-based interface to operate Stretch, which was compatible with the head tracker and mouse setup used for his computer. Throughout the two cycles, multiple improvements were made to the web interface according to Henry’s preferences and performance factors. The web interface had step-size settings to adjust the speed of Stretch’s gripper and arm movement from slowest to fastest. To operate Stretch, Henry would first select either ‘Navigation’ mode to control its base or ‘Manipulation’ mode to control its arm and gripper. Once a mode was selected, Henry clicked on the respective directionality arrows (e.g. extend arm) to initiate Stretch’s movement (Figure 7).

**Figure 8.**
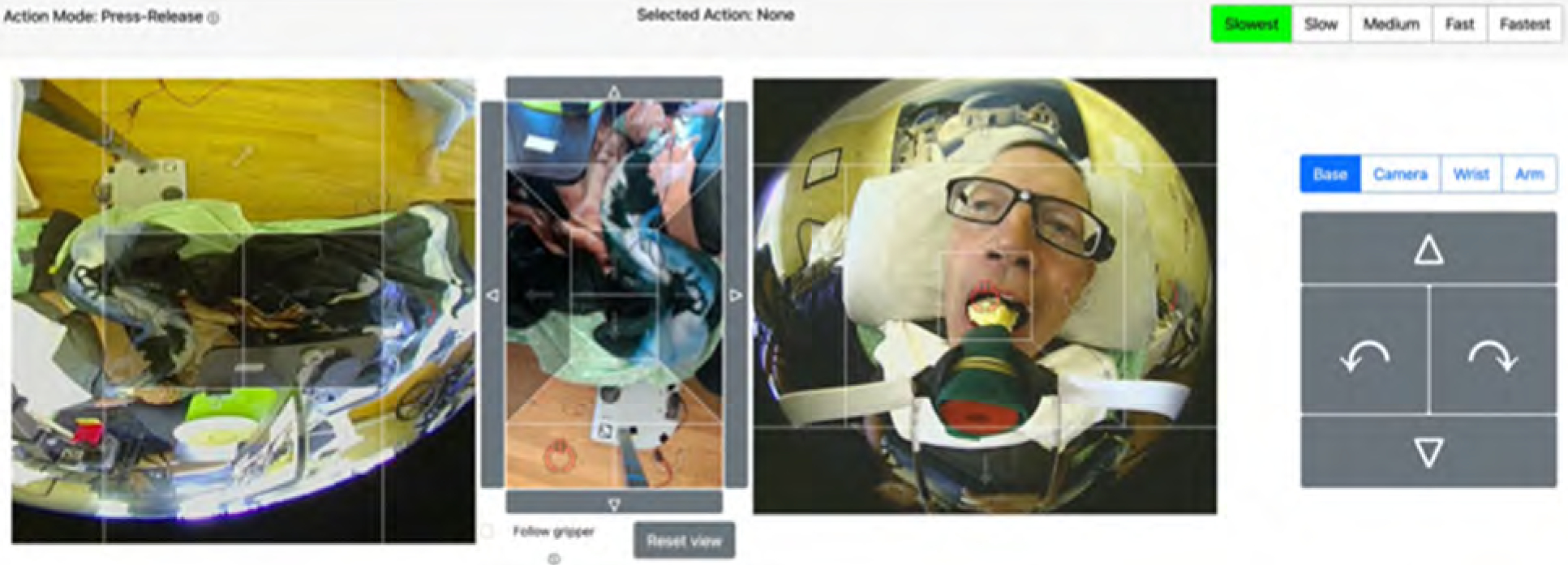
Image of Henry self-feeding with the new button control pad.

However, Henry identified that certain tasks (self-feeding, scratching itches) caused Stretch’s arm to block his left and middle view of his computer screen, obstructing his ability and access to click on Stretch’s controls. Henry collaborated with the engineering team to create a button control pad on the right side of the interface where he had visibility to control Stretch (see Figure 8).

Compared to self-feeding in bed, self-feeding in a wheelchair was more fatiguing for Henry due to increased muscle spasticity, making it difficult for him to use his computer. During these spasms, Henry asked to be tilted back in his wheelchair as a break or asked for his left arm to be propped with a pillow to reduce the likelihood of spasms and increase comfort when doing activities. The OT recommended Henry pace his tasks to prevent episodes of spasms, such as having Henry do activities with fewer performance demands in his bed during the day before being transferred into his wheelchair to do seated tasks, such as playing poker with friends. It was important to Henry not to have spasms amongst his friends when engaging in social activities using Stretch.

### Customising robot functionality to meet user needs

Although Henry liked the customised aspect of Stretch’s web interface, he wanted to automate Stretch’s movement for quicker task performance. In response to this feedback, the engineering team developed a feature for Henry to record and playback Stretch movements to achieve automated behaviours. For example, when turning on a light switch, Henry would orient Stretch in front of his light switch, click on his recorded action labelled as ‘turn on lights’, and Stretch would autonomously flip on his switch. Henry’s interaction with his environment gave him a greater sense of independence without relying on a care partner.

**Figure 9.**
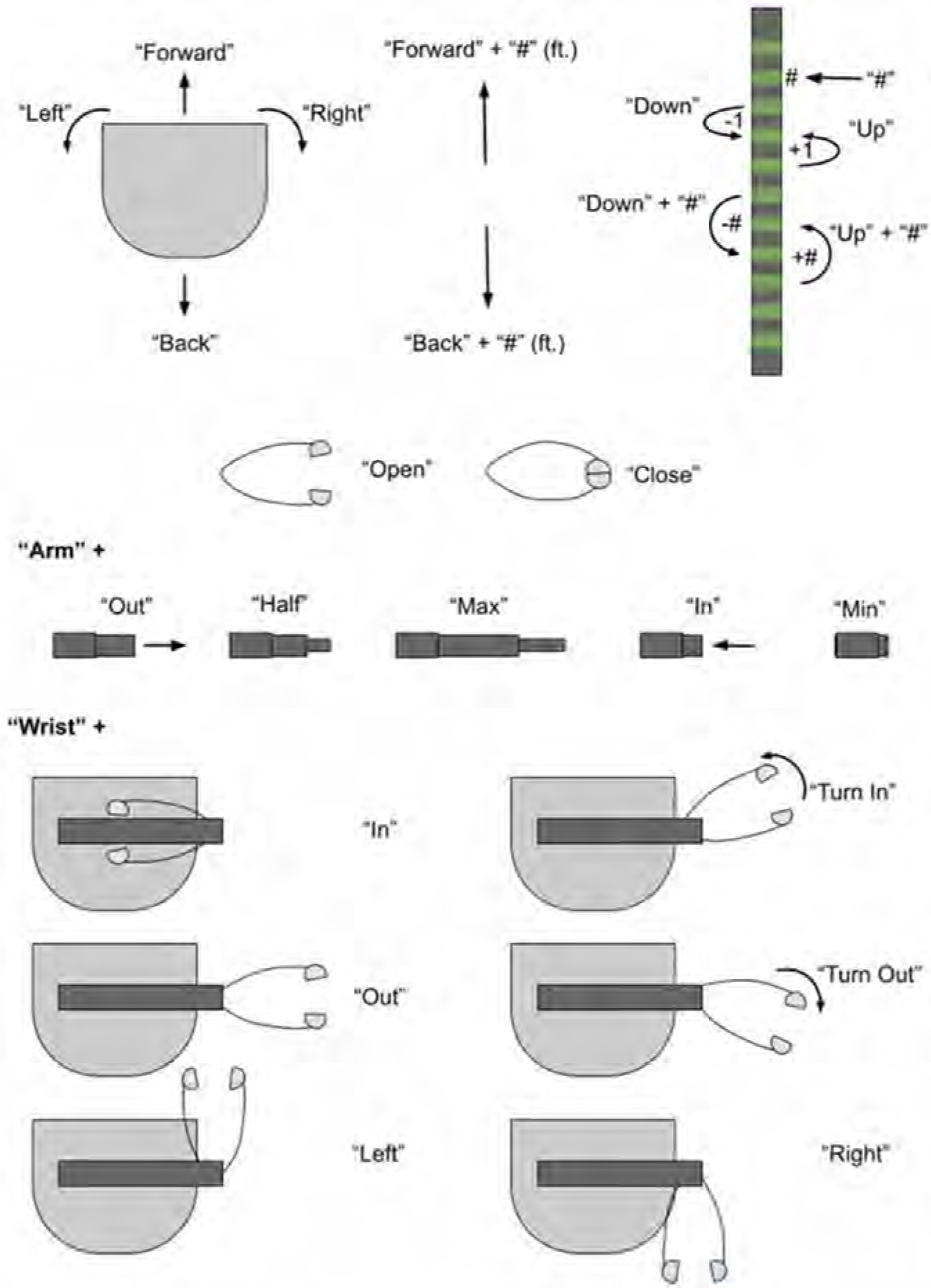
Illustration of Stretch’s voice commands.

Henry and Jane also requested a voice command feature on Stretch for Jane to use since there was no existing way for her to easily operate Stretch. Jane mentioned how ‘[voice commands] are huge because most caregivers are not techies’. She commented, ‘… I’ll be honest, for me to get my laptop and then open it up and log onto this and go onto this site, it’s not going to happen’. The voice commands enabled Jane to navigate Stretch to move in a specific direction: extend, retract, move up and down its arm, and open and close its gripper (Figure 9). She added, ‘I want to take the shortest route possible, and the voice commands allow me’.

### Tool designs

Creating things can be a way to participate in the design process, as tools give us the power to bring our ideas to life (Clemensen et al. 2017). Several tools were designed with Henry and Jane to promote task performance with continual user participation throughout the study.

### Button pusher for percussion vest machine

Henry designed a button pusher tool to start and pause his percussion vest machine when he wanted to take a break from or end the programmed 30-minute session sooner. This provided him the autonomy to manage his comfort without calling for a care partner. It was especially difficult for him to call his care partner when the machine was on due to the loud sound it produced.

### Adaptive spoon and towel holder for self-feeding

**Figure 10.**
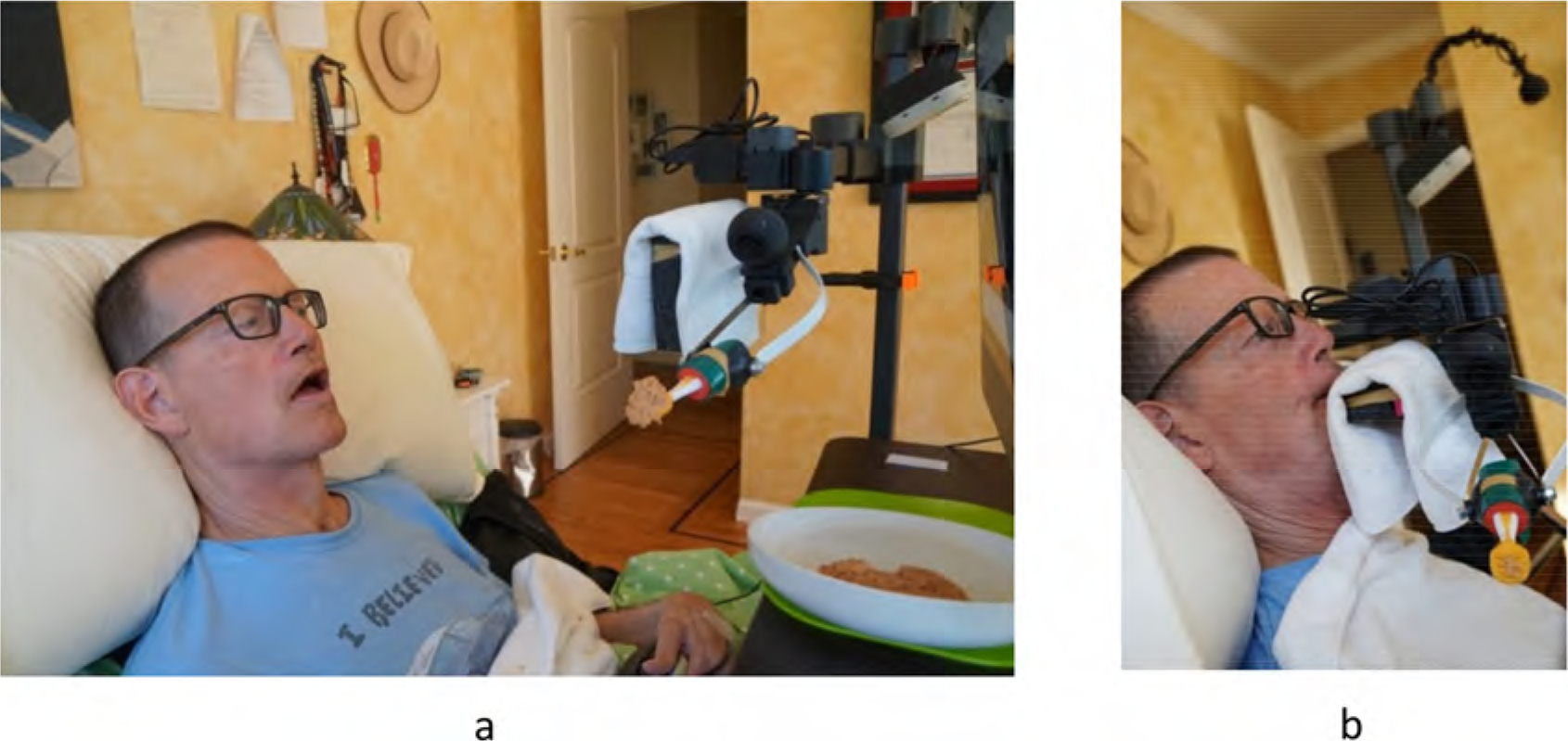
Self-feeding tools and setup. (a) Henry’s self-feeding soft pureed foods with an adapted built-up silicone spoon and scooped plate on top of a Dycem mat. The custom towel holder is accessible in between bites of food for mouth wiping. (b) The foam towel holder was reduced to an inch in length according to Henry’s preferences.

First, the OT self-tested a variety of silicone spoons for self-feeding that were compatible with Henry’s use with Stretch and designed a towel holder made of foam for Henry to wipe his face. Initially, the OT draped a towel over a four-inch-long foam. However, Henry requested a cut to a one-inch length so he could wrap his mouth on the towel for better wiping. The OT set up the environment by putting a Dycem non-slip mat under a scooped-up plate with soft pureed foods and a foam-built-up, flat-designed silicone spoon Henry used to feed himself (Figure 10). This setup enabled Henry to eat with greater independence, as he remarked, ‘I really like eating at my own pace and not being reliant on anyone else’.

### Easy-access playing card holder

**Figure 11.**
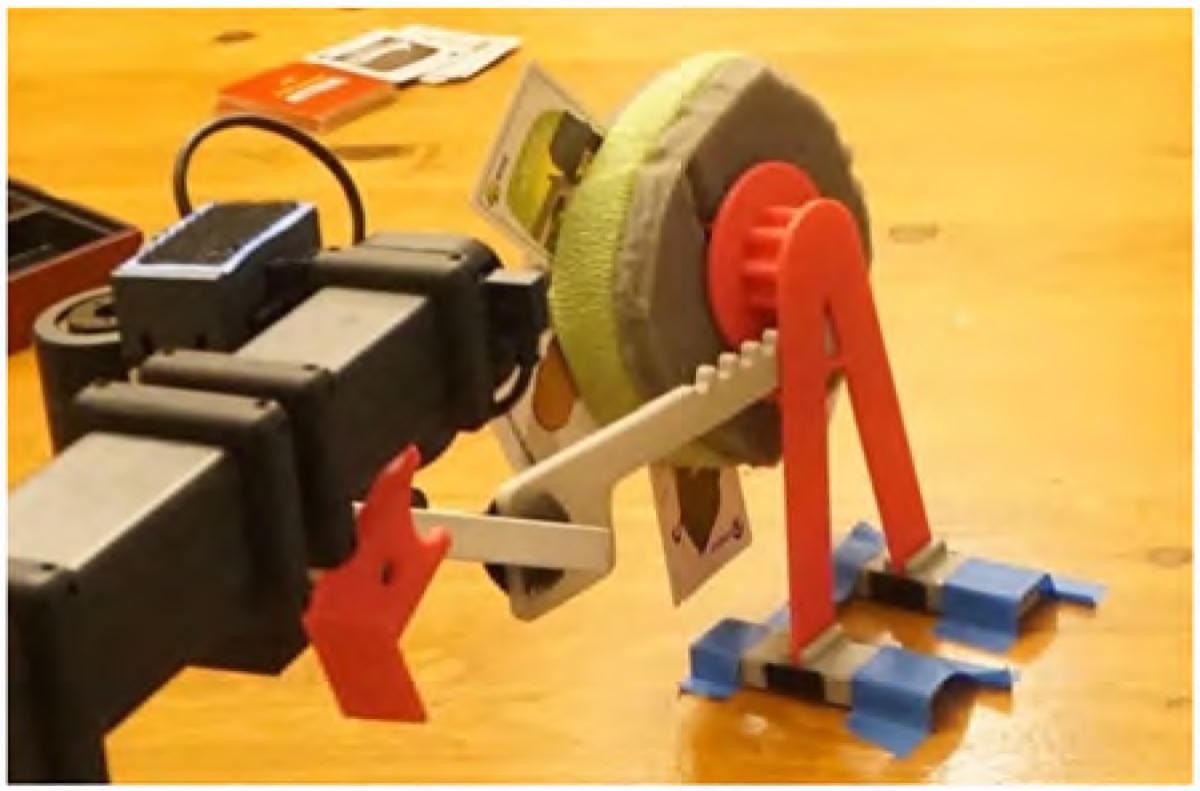
Image of Stretch’s gripper pulling the card holder’s pull tab to rotate the playing cards.

Playing card games is an important social activity for Henry, Jane, their family, and friends. Before Stretch, Henry played on Jane’s or someone else’s team. The OT sliced slots into a foam material where the cards could be inserted, and a contrasting green Coband cohesive medical bandage was added to the slots to increase its visibility so the care partner could distinguish which slots to insert the cards. The robotics engineer designed and 3D printed a spinning playing card holder. The foam material was inserted into the holder, and a pull tab was added. Henry grasped and pulled the pull tab using Stretch’s gripper to rotate foam. This enabled him to position his cards in his preferred position (Figure 11). Henry would cue an opponent to assist him in putting down or inserting cards. With this setup, Henry played poker with Jane and their friends while being on his own team. As Henry describes, ‘I was able to effortlessly play my own hand. The emphasis was poker, not the robot’.

### Arm-mounted tray

**Figure 12.**
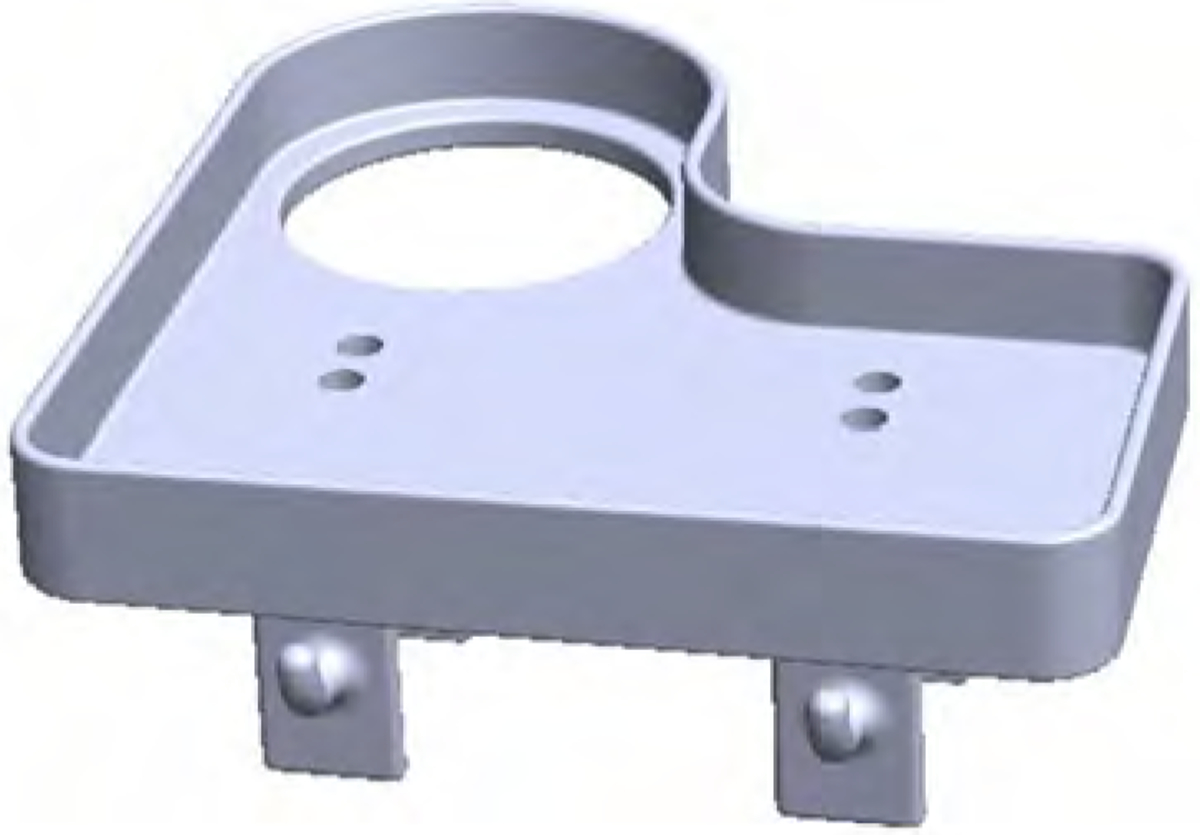
Image of a tray accessory that can be mounted to Stretch’s arm link.

Based on Henry and Jane’s goals to have Stretch support in meal-related tasks, the robotics engineer designed and 3D printed a tray (Figure 12) that clips onto Stretch’s first arm link. This provided a surface for carrying objects, including two hooks in the front and a cup holder to assist with a variety of tasks. Fabric baskets were added to the two hooks for Jane to place additional meal-related items. With this setup, Henry drove Stretch to the kitchen, Jane placed utensils, napkins, and condiments onto the tray, and Henry drove Stretch back to the bedroom. This enabled Jane to make only one trip to the bedroom with their meals and one trip back to the kitchen when they finished their meal since Henry drove Stretch with the used meal items. Together, Henry and Jane collaborated in performing meal-related tasks, instead of Jane partaking solely in this activity and typically making multiple round trips to deliver and clean up meals.

### Barriers to use from the robotics engineer’s and occupational therapist’s perspective

Henry reported how the web interface software still crashes a lot. As a result, Henry and the robotics engineer would often have to restart the interface, which interfered with the natural experience of carrying out a task. Furthermore, navigating Stretch could be challenging. Although the robotics engineer worked on developing ArUco tags to automate Stretch’s positioning to perform activities more precisely and optimally, the accuracy of the tags was unreliable. As a result, Henry positioned Stretch’s base for activities close to his bed or wheelchair (self-feeding, scratching itches). Henry recommended that the base configuration be improved. The robotics engineer also noted that more development is needed to make the motion capture process less demanding and to reduce how much preparatory work goes into every session. The voice commands worked well; however, they required the user to stand close to the speaker and vocalise in an assertive and loud tone. This also needed improvement. The OT was pleased to receive several of the design suggestions from Henry and Jane. She implemented several of these suggestions through multiple iterations and tests with the engineer to meet participant’s needs. ‘Seeing the proactive nature of the participants is a positive sign of a user-centered design approach’, she remarked.

## Discussion

Using immersive participatory design and interdisciplinary collaboration, we illustrated how robotic assistive technologies and other assistive technologies can be improved to support and empower the daily lives of individuals with severe motor impairments and their care partners. Stretch’s features were developed using a participatory design approach and refined through immersive iterative sessions with the users. These resulted in designs that promoted independence and improved the quality of care in meaningful aspects of self-care, productivity, and leisure, as identified by our case study dyad. In this section, we discuss the value of such an immersive participatory design approach in improving assistive robotics and other assistive technologies for older adults. We also discuss insights and strategies beneficial to extending the approach to the design of other assistive robots and technologies that will meet a wider range of older adult needs.

### Value of the immersive participatory design in improving robotic assistive technologies

Exploratory design methods in studies aimed at developing assistive robots for older adults are expected to include a review of older adults’ needs and capabilities. They typically involve observations of how older adults perform their daily living activities and task analyses of these activities, identifying areas where support is needed (Rogers et al., 2020). The positive outcomes of our exploratory research, employing the immersive participatory design approach, have revealed the profound and extensive values that such an approach can offer in supporting the comprehension of researchers and designers in the field of robotics. It presents an opportunity to gather pertinent design feedback from participants who evaluate the system as it progresses through each stage of the iterative design process. The outcome of the approach would emerge from an active engagement of the older adults in the design process to ensure usability and accessibility as recommended (Rogers et al. 2020). This is necessary to enhance the usefulness and application of current robotic technologies in various use case scenarios within the home environment, thereby increasing the potential for wider acceptance of the robots. Assistive robotic technologies have the potential to ease the burdens arising from the ageing population, especially for older adults encountering limitations in performing activities in the home (Beer et al. 2017; Fausset et al. 2011). However, the development must be tuned to fit the needs of the potential users. This is contingent on understanding the use case scenarios, patterns of use, impressions, and perceptions of the users at a deep, holistic level, which can be obtained through immersive participatory design.

The person-centred and goal-oriented approach of the COPM (Law et al. 1990) was beneficial in narrowing down the list of tasks and identifying specific user-centred goals. This was centred around self-care (what an individual needs to do), productivity (what an individual has to do as a contributor to the social and economic community), and leisure (what an individual wants to do to for personal enjoyment and fulfillment). It provided a structured approach to performance assessment in these areas and aligned well with the immersive participatory design process. We consider the COPM a valuable tool for the immersive design approach as it can be tailored to the individuals, with due consideration for their assessment of the level of importance of these goals. The assessment of their level of performance and satisfaction with these goals before and after the intervention with the assistive robot proved helpful in understanding the impact of the intervention. Kerssens et al. (2015) used a similar goal-based outcome measure to identify the most pressing needs of home-dwelling older adults with and without cognitive impairment. This approach leads to personalised technology that promotes positive engagement.

This is a case study with a couple, so the end products will not be generalised to everyone in the target audience of older adults. Henry and Jane Evans have their unique characteristics, experiences, and environmental contexts. However, the participatory design process used in this study can be adopted while co-designing with a wider range of older adults. Fusing the immersive experience with the traditional participatory design helped to gain a firsthand, in-depth understanding of the unique characteristics of the target end user, their environmental context, and their level of performance. When designers have a firsthand understanding of the user’s context and characteristics, their codesign process with the end user can better ensure the design fits critical user needs. This approach was exemplified in the experience of the OT and robotics engineer who lived in Henry and Jane Evans’ home for two cycles of two weeks each. Our research team found that the exchange of findings and revision of prototypes proved effective in the continuous feedback loop that led to the improved version of the robot and the interfaces. Furthermore, this immersive experience allowed researchers to richly consider the interaction between the dyad’s extrinsic factors (i.e. social support and systems, culture, value, natural environment, build environment and technology,) and the influence Stretch has in supporting their everyday lives. Subsequently, the study demonstrated Stretch’s ability to promote Henry and Jane’s performance and satisfaction in the meaningful activities they engaged in individually and together.

### Limitations of the approach

Limitations of the approach include the logistics of being in the same environment as the user for an extended period, as we described. However, adaptations of this immersive participatory design process can be created to give researchers the opportunity to learn more about the users and their environment. A shorter period in the users’ environment or a retreat together to discover use-case scenarios are feasible adaptations that could work in other contexts. Simulated home environments can also be a useful proxy setting for immersive participatory design sessions.

Also, the importance of educating the users to improve their technical capacity with the robot (or technology) cannot be overemphasised. Henry and Jane had considerable time with the researchers to learn how to use the technology and to try it on their own with ongoing support. In situations where extended periods for user learning, testing, and independent trial may not be feasible, adaptations such as educational materials and training modules would be beneficial. The immersive method also provided an opportunity for the research team to build a strong relationship with the users, which may not always be available in other contexts. We encourage researchers to invest time in developing such relationships through alternative means, for example, through video conversations or visits before the study to better understand the users.

### Insights for extending the approach to other older adult populations and assistive technologies

Several design features implemented were based on feedback obtained from the immersive participatory design strategy as we worked towards achieving the task goals for Henry and Jane. While the design implementations were centred around their specific needs, some of the design themes can be extended to other older adult populations with varying age groups and abilities. We share some of these themes in the form of recommendations as follows:

User-driven design: this provides the opportunity for users to control the technology in the way they prefer for selected actions. The immersive participatory design technique lets users and the team understand the problem space collaboratively, think about challenges, and explore and creatively design solutions. This approach made users co-designers throughout the entire design process, ensuring that their activity needs and preferences were represented in the resultant robot designs. The resulting designs were tailored to be fit, safe, intuitive, and comfortable for the user. Users felt more fulfilled being part of the design process, experiencing benefits such as the ability to carry out customisations through the interface to meet their needs. For instance, in our case study, users were trained and proficient in planning the robot motion sequence to perform specific repetitive tasks automatically. This provided users with various options to customise the technology for the automation of their tasks. Users were generally more content using the resultant design because they were fully invested in the design process, which was structured around their perspective on the product. This facilitated satisfaction, willingness to use, and actual use of the product, reducing the possibility of technology abandonment in the long term.

Alternative means of control: the robot was co-designed with users to have different control options (redundancy in design) to cater to their needs. To illustrate this, a voice-based interface was incorporated for the care partner, who did not have the physical space or time to use a screen-based interface while caring for the care recipient. On the other hand, the care recipient controlled the robot through the screen-based interface because he could not use voice commands. Other control options include a joystick or sip-and-puff device for individuals who cannot use a mouse or an eye-tracking device for persons with limited neck mobility. This redundancy in control mechanisms can be extended to other assistive robots and technology to accommodate the peculiarities of diverse users. The feedback provided by the robot was also adapted to suit the couple based on their use patterns and preferred feedback content.

Agile, iterative development: this immersive participatory design approach inspired empathy and spurred creativity in developments, as seen in the tool designs made to fit various needs in the home in the context of our case study. The value in this extends to the shortening of the design-prototype-evaluate-redesign iteration loop, as the researchers, designers, and potential users are all co-located and immersed in the same environment. This also enhances the flexibility of the design, making it easier to adapt the robot for various uses, including the incorporation of other tools (such as those used for the percussion vest machine, laundry tasks, and meal setup/clean-up). This provides the option for the robot to be adapted to various uses based on the user’s needs and task demands in a very short time span.

In conclusion, reflecting on this approach for designing other assistive technologies for older adults, we find value in understanding the environment, context, and task constraints of the users. This approach inspires design implementations that meet users’ needs, enhance their abilities, and address their concerns. We recommend this form of immersive participatory design for other assistive technologies and developments. There are higher chances of design outcomes resulting in technologies that better address and adapt to user needs, which may often change with time and circumstances. Such technologies can be instrumental in holistically improving the quality of life for a wider range of older adults across different age groups and abilities.

## Figures and Tables

**Table 1.** Distinguishing immersive participatory design from other techniques.

Levels	setting	End-user’s input	Iterative	co-Design with user

One-time user testing	controlled setting	Yes	No	No
Iterative user testing		Yes	Yes	No
Participatory design		Yes	Yes	Yes
Immersive one-time user testing	User’s environment	Yes	No	No
Immersive iterative user testing		Yes	Yes	No
Immersive participatory design		Yes	Yes	Yes

## Practitioners’ Summary

- An occupational therapist and robotics engineer embedded themselves in the home of an older adult couple and used an immersive participatory design for customising a mobile robot.
- The robot supported them in everyday activities and enhanced their social-emotional well-being.
- Co-designing with older adults can generate solutions for better ageing in place.

## Appendix

**Table:** Canadian Occupational Performance Measure (COPM) Goals, Associated Activity for Each Participant, and Anecdotal Impressions.

Participant	COPM Goal	Description of activity performed using Stretch	Anecdotal/Narrative impressions

Henry Evans	Self-feeding	Self-feeding soft pureed foods for breakfast and dessert using an automated, motion-sequence capture feature.	‘The automation is great because it makes the feeding device practical’.
	Maintaining comfort	Operating percussion vest machine to clear airways with a button tool customised tool. Scratching itches on Henry’s face and head with a customised hairbrush tool.	‘Being able to pause my programmed 30-minute session on my percussion vest machine is important for my comfort because it’s hard to call for a caregiver to help when they are someplace else. Plus, they cannot hear me (non-verbal vocalizations) because the machine is too loud’. ‘I experience 3–4 violent, debilitating itches on my head and face every hour and rely on a caregiver to help me. Scratching using Stretch gives me instant relief’.
	Flipping a light switch	Turning on/off bedroom light switch using recorded automated movements.	‘I have to be honest; while automating the on/off switch for the arm was very empowering, the fact that the automated base movement didn’t work for this function was disheartening because the precise base location was critical to being able to operate the switch again once you had moved the robot to do something else’.
	Meal preparation and clean-up	Assist Jane in transporting meal-related items from the kitchen to Henry’s bedroom and back.	‘It was great to be able to help Jane in a meaningful way. It made me feel useful again.
	Social participation via card games	Playing poker with friends using customised card holder.	‘The best part about the poker experience was the fact that operating the robot was not a distraction to anyone because it was very natural and real-time.
Jane Evans	Participating in physical exercise	Engaging in routine exercise (which we dubbed ‘Stretchercise’) using a customised web interface.	‘I can absolutely see myself logging into the Stretchercise, then initiating the exercise. It’s very cool. Very cool. It’s easy. I don’t feel like it’s a million of steps to do’.
	Assistance in meal-related tasks	Reduce travel time from the kitchen to Henry’s bedroom to deliver meals and clean up meals.	‘It was amazing. It gets kind of lonely doing it by yourself, and then he (Henry) would be on YouTube or would be watching TV or whatever, and you know, it gets old after a while. And not that I have to have somebody hold my hand 24 hours, but it’s nice to work together. It really is’.
	Household cleaning	Floor cleaning.	Henry said, ‘Swiffering a bust [ineffective, Roomba better]’.
	Laundry assistance	Henry is towing a laundry hamper near laundry room.	‘Well, I envisioned when he when he brings over the laundry basket that I could be or would be cooking or whatever, and then I see the robot come down and stop at that laundry door with the things. I mean, it’s huge [help]; it’s right there. Basically, I’d detach it from the robot, bring it right up to the machine, and load’.
	Social participation with Henry	Play a game with Henry.	‘It’s wonderful seeing him play cards on his own team and the robot being in the background and not the centre of attention’.
